# Detection of Sulfur-Fumigated Paeoniae Alba Radix in Complex Preparations by High Performance Liquid Chromatography Tandem Mass Spectrometry

**DOI:** 10.3390/molecules17088938

**Published:** 2012-07-26

**Authors:** Jie Wu, Hong Shen, Jun Xu, Ling-Ying Zhu, Xiao-Bin Jia, Song-Lin Li

**Affiliations:** 1 Department of Pharmaceutical Analysis and Metabolomics, Jiangsu Province Academy of Traditional Chinese Medicine, Nanjing 210028, China; 2 Key Laboratory of New Drug Delivery System of Chinese Materia Medica, Jiangsu Province Academy of Chinese Medicine, Nanjing 210028, China

**Keywords:** sulfur-fumigation, Paeoniae Alba Radix, *Paeonia lactiflora*, complex preparations, HPLC-TQ-MS/MS, multiple reaction monitoring (MRM)

## Abstract

Detection of sulfur-fumigated Paeoniae Alba Radix (PAR) in different complex preparations is challenging due to the relatively lower content of PAR and interference from more complicated components in complex preparations with different multiple constituent herbs. In this study, a high performance liquid chromatography- triple-quadrupole tandem mass spectrometry method was developed for detecting sulfur-fumigated PAR in different complex preparations. Paeoniflorin, the major component of PAR, and paeoniflorin sulfonate, the characteristic artifact transformed from paeoniflorin during sulfur-fumigation of PAR, were used as chemical markers. Multiple reaction monitoring (MRM) scan was employed to maximize sensitivity and selectivity. Through optimizing full mass scan and daughter ion scan conditions, two mass transitions were selected and employed respectively for unequivocal identification of paeoniflorin and paeoniflorin sulfonate. The detection limits for paeoniflorin and paeoniflorin sulfonate using MRM were much lower than those detected with UV 270 nm. Paeoniflorin and paeoniflorin sulfonate could be simultaneously detected in different commercial PAR-containing complex preparations without interference of other components using the established method, indicating that the newly established method was selective and sensitive enough for screening sulfur-fumigated PAR in commercial complex preparations.

## 1. Introduction

Paeoniae Alba Radix (PAR), derived from the root of *Paeonia lactiflora*, is a commonly used medicinal herb with claims of antispasmodic, tonic, astringent and analgesic properties [1]. In Traditional Chinese Medicine, this herb is prescribed as the major constituent material of many complex preparations [2,3,4,5].

PAR was recently reported as being sulfur-fumigated during post-harvest handling to keep moist, preserve color, and prevent insects and moulds [6,7,8]. Accumulated studies showed that sulfur-fumigation can induce chemical transformation of paeoniflorin, the main bioactive component of PAR, into its artifact paeoniflorin sulfonate [6,8,9,10,11], and consequently alter the bioactivities [11] and pharmacokinetics [12] of PAR. As a matter of fact, sulfur-fumigated medicinal herbs have been regarded as drugs of inferior quality by the State Food and Drug Administration of China (SFDA) since 2004 [13]. Therefore, screening for sulfur-fumigated PAR and sulfur-fumigated PAR-containing complex preparations is very important for the effective and safe application of PAR-containing complex preparations.

High performance liquid chromatography-ultraviolet or mass spectrometry (HPLC-UV or MS) methods using paeoniflorin sulfonate as chemical marker [7,11,14,15] were developed for identification of sulfur-fumigated PAR raw material. However, to the best of our knowledge, no study on screening for sulfur-fumigated PAR in complex preparations was reported. 

It is well known that complex herbal preparations are composed of different kinds of herbs, and different complex preparations have different compositions and proportions of constituent herbs. Practically, when LC-UV was used to identify sulfur-fumigated PAR in complex preparations, the more complicated constituents of different complex preparations may interfere with the separation of target analytes due to the poor selectivity of the UV detector, and consequently it is difficult to develop a universal LC-UV method for the identification of sulfur-fumigated PAR in different complex preparations. Furthermore, UV detectors might not sensitive enough for target analytes when the content of PAR in the complex preparations was relatively low. 

High performance liquid chromatography triple-quadrupole tandem mass spectrometry (HPLC-TQ-MS/MS) has advantages of ion fragmentation and many scan modes, such as selective ion recording (SIR), multiple reaction monitoring (MRM), which are helpful for not only identity assignment through rationalization of ion fragments, but also sensitivity improvement and selectivity enhancement through MRM scan mode [15,16].

In this study, using HPLC-TQ-MS/MS with two mass transitions MRM scan, a sensitive and selective method to screen sulfur-fumigated PAR in complex preparations was developed, and was successfully applied for analysis of commercial PAR-containing complex preparations with different constituent herbs.

## 2. Results and Discussion

### 2.1. Identification of Paeoniflorin and Paeoniflorin Sulfonate in Self-Prepared Sulfur-Fumigated PAR by Full Mass Scan

**Figure 1:**
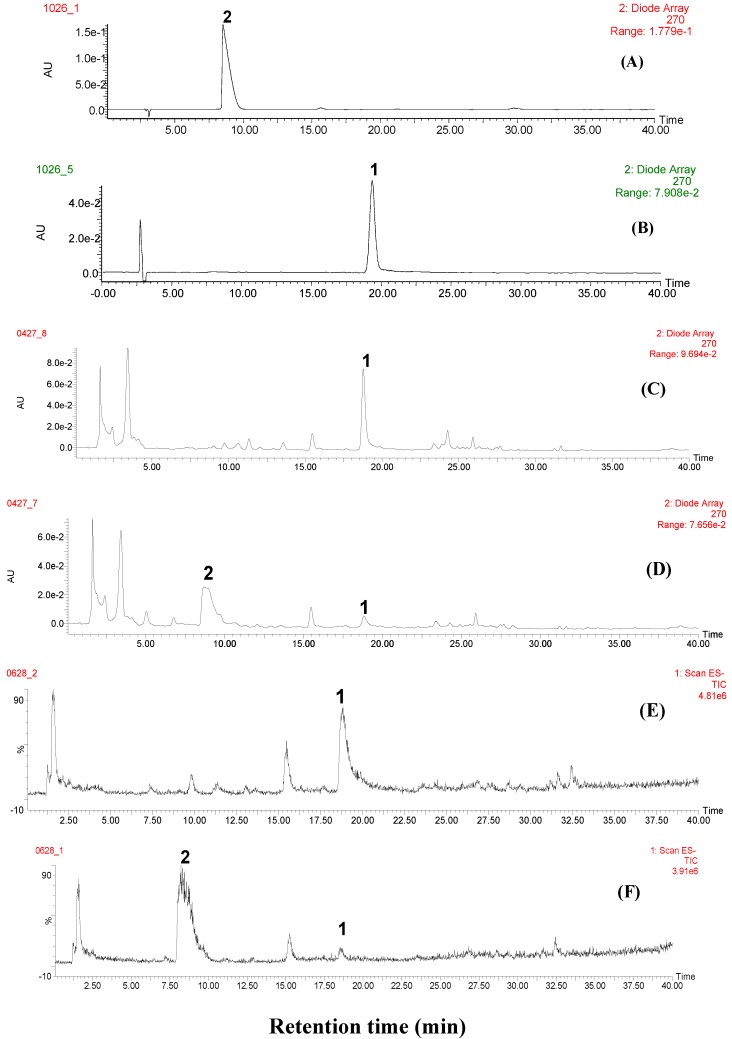
Representative chromatograms of marker compounds and PAR. (**A**) marker compound paeoniflorin sulfonate; (**B**) marker compound paeoniflorin; (**C**) non-fumigated PAR (JPACM-01-01) monitored at UV 270 nm; (**D**) sulfur-fumigated PAR (JPACM-01-02) monitored at UV 270 nm; (**E**) TIC of non-fumigated PAR (JPACM-01-01); (**F**) TIC of sulfur-fumigated PAR (JPACM-01-02); **1**: paeoniflorin; **2**: paeoniflorin sulfonate.

Non-fumigated and sulfur-fumigated PAR samples were comparatively analyzed by HPLC-PDA-TQ-MS/MS. Figure 1A–F show the representative UV and full scan total ion chromatograms (TICs) of paeoniflorin, paeoniflorin sulfonate and PAR samples.

**Figure 2:**
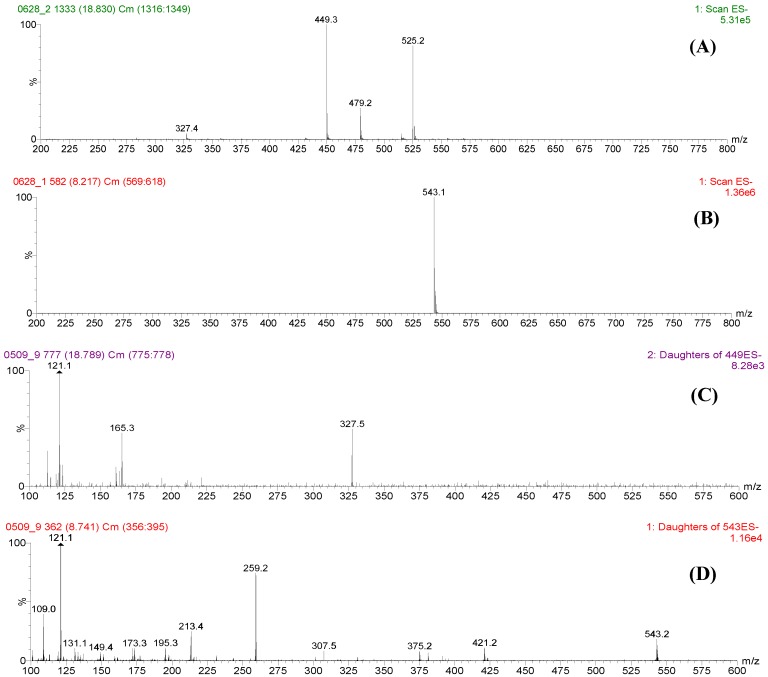
Mass spectra of paeoniflorin and paeoniflorin sulfonate. (**A**) full mass scan of paeoniflorin; (**B**) full scan of paeoniflorin sulfate; (**C**) daughter ion scan of paeoniflorin (parent ion *m/z* 449); (**D**) daughter ion scan of paeoniflorin sulfonate (parent ion *m/z* 543).

From Figure 1C,D it was found that compared with that in non-fumigated sample, the peak height of the main component (peak 1, t_R_ = 18.8 min) was significantly decreased in sulfur-fumigated samples, whereas a new compound (peak 2, t_R_ = 8.7 min) was detected in the sulfur-fumigated sample. Different cone voltages were tested to optimize ionizing conditions for peak 1 and peak 2 and it was found that the optimal voltages for peak 1 and peak 2 were 25 V and 40 V, respectively. The mass spectra of peak 1 and peak 2 under the chosen conditions are shown in Figure 2A,B.

Peak 1 was proposed to be paeoniflorin. In the mass spectrum of peak 1, four ions were observed, they were rationalized to be the formic acid adduct molecular ion *m/z* 525 [M+HCOOH-H]^−^, molecular ion *m/z* 479 [M-H]^−^, and fragment ions *m/z* 449 [M-H-30]^−^, and *m/z* 327 [M-H-30-122]^−^ of paeoniflorin. This deduction was further confirmed by comparing the retention time and mass spectrum with that of paeoniflorin marker compound. In the mass spectrum of peak 2, only one ion was found, it was deduced to be molecular ion *m/z* 543 [M-H]^−^ of paeoniflorin sulfonate, thus peak 2 was assigned to be paeoniflorin sulfonate, which was also confirmed by comparing the retention time and mass spectrum with that of paeoniflorin sulfonate reference compound.

### 2.2. Fragmentation of Paeoniflorin and Paeoniflorin Sulfonate by Daughter Ion Scan

To extensively optimize the conditions for fragmentation of paeoniflorin and paeoniflorin sulfonate, the samples were further analyzed with daughter ion scan, with ion *m/z* 449 and *m/z* 543 selected as parent ions of paeoniflorin and paeoniflorin sulfonate respectively. Different collision voltages were tested to optimize fragmentation conditions. It was found that 15 V and 40 V were suitable for fragmentation of ions *m/z* 449 and *m/z* 543, respectively. The typical mass spectra of these two parent ions were shown in Figure 2C,D.

**Figure 3:**
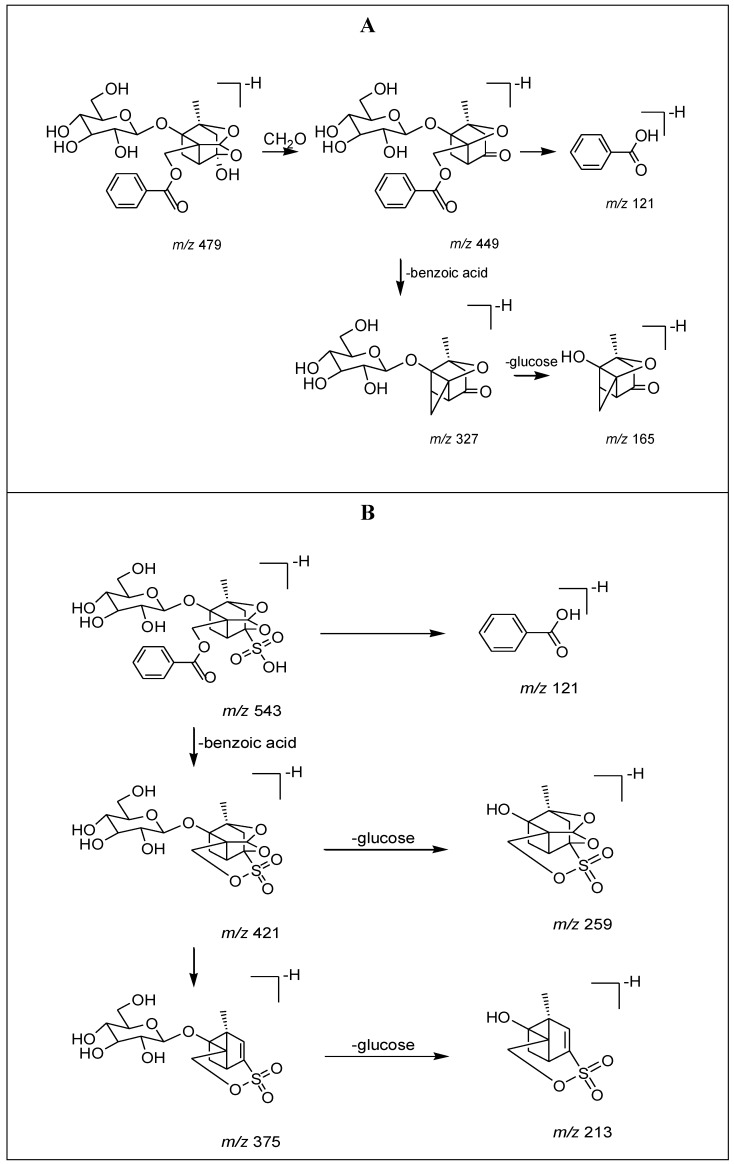
Rationalization of fragments. (**A**) paeoniflorin; (**B**) paeoniflorin sulfonate.

From Figure 2, it was found that ion *m/z* 449 could be further fragmented into ions *m/z* 327, 165 and 121, while ion *m/z* 543 could be further fragmented into ions *m/z* 421, 375, 259, 213 and 121. The assumed fragmentation pathways for these two compounds were illustrated in Figure 3.

### 2.3. Selectivity and Sensitivity by MRM Scan

**Figure 4:**
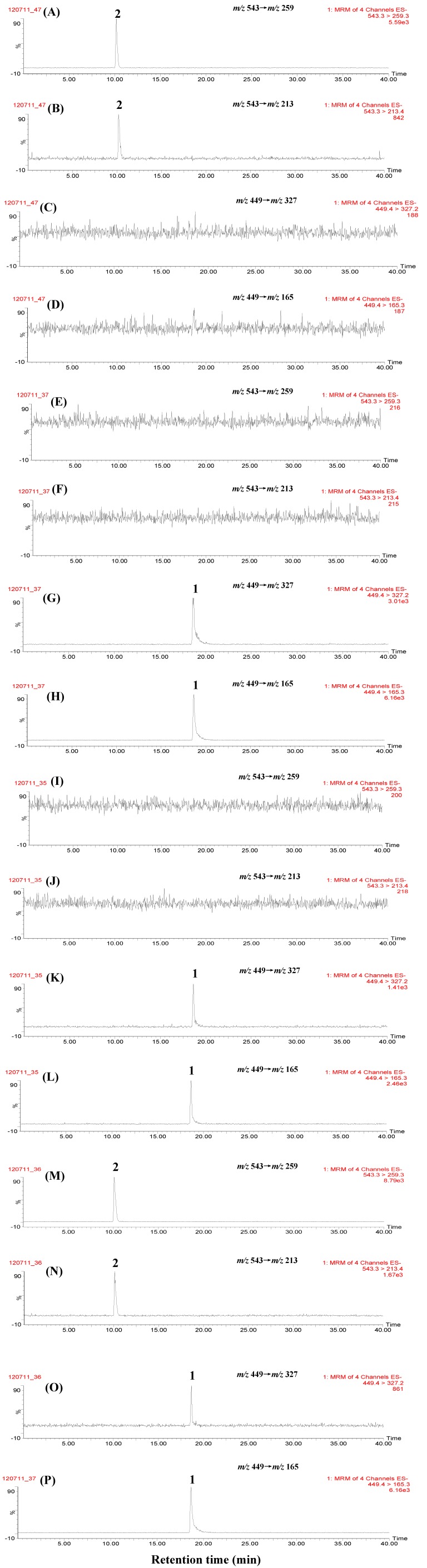
Representative chromatograms of marker compounds and PAR by MRM scan. (**A**) paeoniflorin sulfonate ion transition *m/z* 543→*m/z* 259; (**B**) paeoniflorin sulfonate ion transition *m/z* 543→*m/z* 213; (**C**) paeoniflorin sulfonate ion transition *m/z* 449→*m/z* 327; (**D**) paeoniflorin sulfonate ion transition *m/z* 449→*m/z* 165; (**E**) paeoniflorin ion transition *m/z* 543→*m/z* 259; (**F**) paeoniflorin ion transition *m/z* 543→*m/z* 213; (**G**) paeoniflorin ion transition *m/z* 449→*m/z* 327; (**H**) paeoniflorin ion transition *m/z* 449→*m/z* 165; (**I**) non-fumigated PAR (JPACM-01-01) ion transition *m/z* 543→*m/z* 259; (**J**) non-fumigated PAR (JPACM-01-01) ion transition *m/z* 543→*m/z* 213; (**K**) non-fumigated PAR (JPACM-01-01) ion transition *m/z* 449→*m/z* 327; (**L**) non-fumigated PAR (JPACM-01-01) ion transition *m/z* 449→*m/z* 165; (**M**) fumigated PAR (JPACM-01-02) ion transition *m/z* 543→*m/z* 259; (**N**) umigated PAR (JPACM-01-02) ion transition *m/z* 543→*m/z* 213; (**O**) fumigated PAR (JPACM-01-02) ion transition *m/z* 449→*m/z* 327; (**P**) fumigated PAR (JPACM-01-02) ion transition *m/z* 449→*m/z* 165. 1: paeoniflorin; 2: paeoniflorin sulfonate.

It is well known that in LC-TQ-MS/MS analysis, two mass transition MRM scans have more selectivity and the best sensitivity among all scan modes, thus two mass transitions, *i.e.*, *m/z* 449→*m/z* 327 and *m/z* 449→*m/z* 165 for paeoniflorin, *m/z* 543→*m/z* 259 and *m/z* 543→*m/z* 213 for paeoniflorin sulfonate were monitored, respectively, in the MRM scans. Figure 4 shows the MRM chromatograms of marker compounds, non-fumigated and self-prepared sulfur-fumigated PAR samples. It was found that paeoniflorin (**1**) and paeoniflorin sulfonate (**2**) were simultaneously detected in sulfur-fumigated PAR samples, and no other peaks interfered with the detection of these two marker compounds, suggesting that MRM scan had higher selectivity for the identification of paeoniflorin and paeoniflorin sulfonate in sulfur-fumigated PAR. Furthermore, the ion intensity ratios of two mass transitions for reference pure paeoniflorin sulfonate (*m/z* 543→*m/z* 259 *vs. m/z* 543→*m/z* 213) and paeoniflorin (*m/z* 449→*m/z* 327 *vs.m/z* 449→*m/z* 165) were calculated to be 6.53 and 0.49 respectively. 

As far as sensitivity was concerned, the limit of detection (LOD) by MRM scan and UV/270nm detection were compared. It was found that under the present conditions, the LODs by MRM scan were tested to be 0.21 ng/mL (*m/z* 449→*m/z* 327) and 0.53 ng/mL (*m/z* 543→*m/z* 259) for paeoniflorin and paeoniflorin sulfonate respectively, while LODs by UV detection were generally at “µg/mL” orders of magnitude [7,14], for example, 0.11 µg/mL and 0.27 µg/mL for paeoniflorin and paeoniflorin sulfonate, respectively, tested by UV detection (270 nm) in this study, suggesting that the established MRM scan was much more sensitive than UV for the detection of paeoniflorin and paeoniflorin sulfonate in sulfur-fumigated PAR. In addition, it is worth mentioned that the newly established method were also more sensitive than the recently published HPLC-MS approach, in which MRM scan was also employed with LODs of 6–8 ng/mL for screening sulfonate derivatives in PAR [15].

### 2.4. Detection of Paeoniflorin Sulfonate in Water Decoction of Sulfur-Fumigated PAR

It is well known that water decoction is not only the major form of folk medication, but also the commonly used extraction method for complex preparations in traditional Chinese medicines [17,18,19]. Decocting may induce chemical alterations of bioactive components in medicinal herbs [20]. Therefore, water decoction and 50% methanol extract of sulfur-fumigated PAR sample (JPACM-01-02) were compared to see whether or not decocting can cause any significant chemical changes to paeoniflorin and paeoniflorin sulfonate. It was found that paeoniflorin and paeoniflorin sulfonate could also be detected in water decoction of sulfur-fumigated PAR sample (data not shown), suggesting that the established method was also applicable for screening sulfur-fumigated PAR-containing complex preparations produced by water extraction.

### 2.5. Analysis of Commercial PAR Samples and PAR-containing Complex Preparations

**Figure 5:**
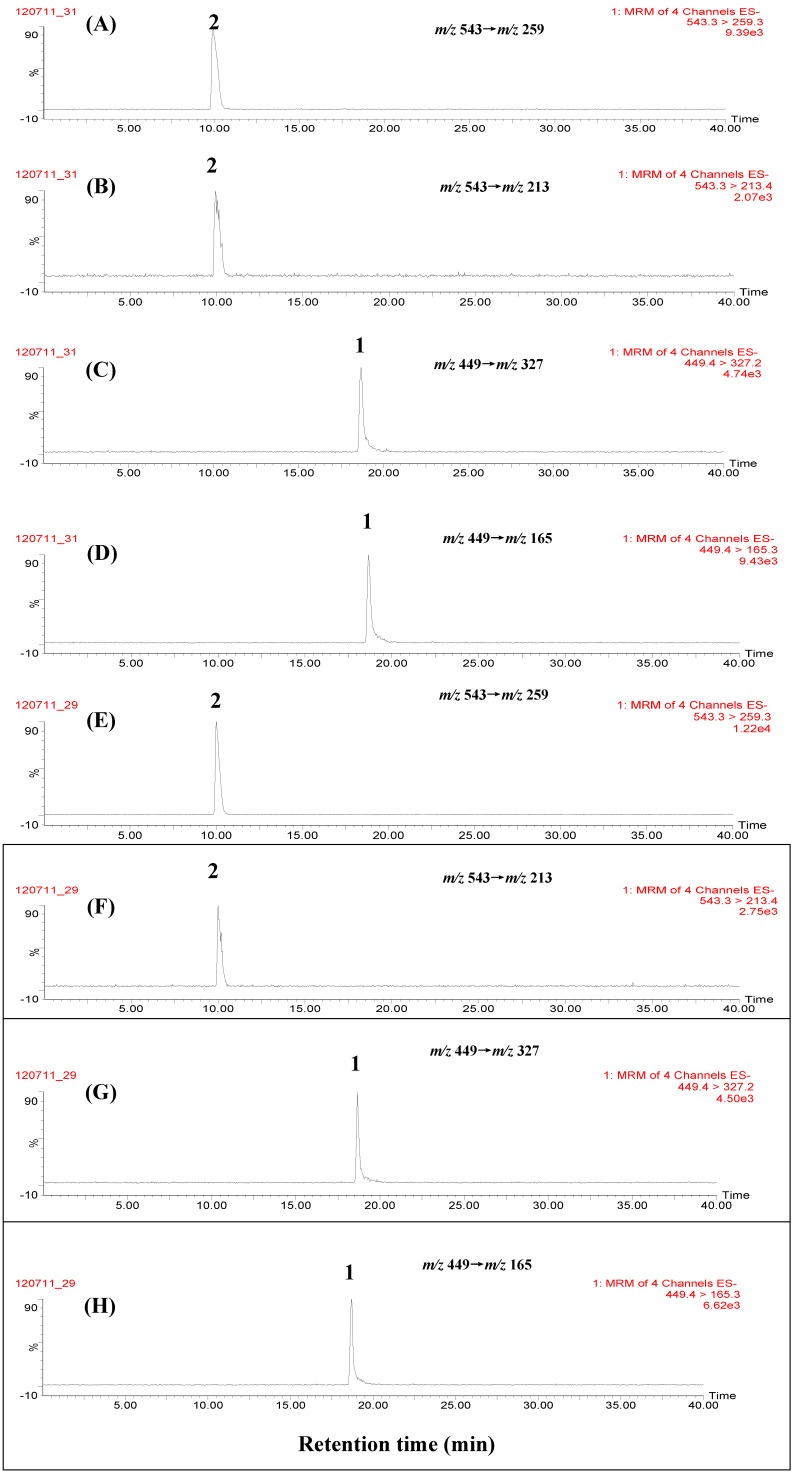
Representative chromatograms of different PAR-containing complex preparations by MRM scan. (**A**) XYW (JPACM-02-06) ion transition *m/z* 543→*m/z* 259; (**B**) XYW (JPACM-02-06) ion transition *m/z* 543→*m/z* 213; (**C**) XYW (JPACM-02-06) ion transition *m/z* 449→*m/z* 327; (**D**) XYW (JPACM-02-06) ion transition *m/z* 449→*m/z* 165; (**E**) MRW (JPACM-02-05) ion transition *m/z* 543→*m/z* 259; (**F**) MRW (JPACM-02-05) ion transition *m/z* 543→*m/z* 213; (**G**) MRW (JPACM-02-05) ion transition *m/z* 449→*m/z* 327; (**H**) MRW (JPACM-02-05) ion transition *m/z* 449→*m/z* 165. 1: paeoniflorin; 2: paeoniflorin sulfonate.

Seventeen commercial PAR samples (JPACM-01-07 to JPACM-01-23) and seven PAR-containing complex preparations with different constituent herbs were next analyzed using the established method. The representative chromatograms are shown in Figure 4 and Figure 5, and the results are summarized in Table 1. It was found that paeoniflorin and paeoniflorin sulfonate were simultaneously detected in sixteen of seventeen commercial PAR samples and all seven PAR-containing complex preparations collected, and no other peaks interfered with the detection of these two compounds even in seven different complex preparations. Further more, the ion intensity ratios of two mass transitions for paeoniflorin sulfonate (*m/z* 543→*m/z* 259 *vs. m/z* 543→*m/z* 213) and paeoniflorin (*m/z* 449→*m/z* 327 *vs.m/z* 449→*m/z* 165) were calculated to be 6.52 ± 0.29 (RSD 4.4%) and 0.50 ± 0.048 (RSD 9.6%) respectively in seventeen raw materials, whereas 5.55 ± 0.52 (RSD 9.3%) and 0.53 ± 0.03 (RSD 6.2%) respectively in seven complex preparations, suggesting that there were a little matrix effects on ionization of two marker compounds in raw materials and complex preparations. All these results suggested that the established method had high selectivity for the identification of sulfur-fumigated PAR, and is a universal method for screening sulfur-fumigated PAR in complex preparations with different constituent herbs.

It was also found from Table 1 that paeoniflorin sulfonate was widely detected in the investigated PAR samples and its complex preparations collected from different localities and producers. So it could be concluded that sulfur-fumigation is currently still a common process method in post-harvest handling of PAR. Therefore it is recommended that SFDA of China should strengthen the enforcement to prohibit PAR being sulfur-fumigated, so that PAR-containing complex preparations could be effectively and safely used in clinical medications.

**Table 1:** Detection of paeoniflorin sulfonate in PAR and PAR-containing complex preparations.

PAR			
Sample code	Collection locality	Collection time (year-month)	Result
JPACM-01-01	Bozhou, Anhui province	2009-01	−
JPACM-01-02	Bozhou, Anhui province	2009-01	+
JPACM-01-03	Shangqiu, Henan province	2009-01	−
JPACM-01-04	Shangqiu, Henan province	2009-01	+
JPACM-01-05	Jiang county, Shanxi province	2009-01	−
JPACM-01-06	Jiang county, Shanxi province	2009-01	+
JPACM-01-07	Bai Xin Pharmacy, Nanjing	2009-10	+
JPACM-01-08	Bao Feng Tai Ping Pharmacy, Nanjing	2009-10	+
JPACM-01-09	Hua Yue Pharmacy, Nanjing	2009-10	+
JPACM-01-10	Lao Bai Xing Pharmacy, Nanjing	2009-10	+
JPACM-01-11	Lao Bai Xing Pharmacy, Nanjing	2009-10	+
JPACM-01-12	Xian Sheng Pharmacy, Nanjing	2009-10	+
JPACM-01-13	Xian Sheng Pharmacy, Nanjing	2009-10	+
JPACM-01-14	Zhi Lin Pharmacy, Nanjing	2009-10	+
JPACM-01-15	Tian Shi Pharmacy, Nanjing	2009-10	+
JPACM-01-16	Hong Ji Tang Pharmacy, Jinan	2010-02	+
JPACM-01-17	Jian Lian Pharmacy, Jinan	2010-02	+
JPACM-01-18	Shen Nong Ben Cao Pharmacy, Jinan	2010-02	+
JPACM-01-19	Qi Lu Yi Kang Pharmacy, Jinan	2010-02	+
JPACM-01-20	Bozhou Chinese Yinpian company, Bozhou	2009-11	+
JPACM-01-21	Bozhou county, Anhui province	2009-11	−
JPACM-01-22	Bozhou county, Anhui province	2009-11	+
JPACM-01-23	Fu Shun Pharmacy, Liaoning province	2010-02	+
**PAR-containing complex preparations**			
**Sample code**	**Preparation names (herbs contained)**	**Producer **	**Result**
JPACM-02-01	SJWTKL^ *^ (Evodiae Radix, Murrayae Folium et Cacumen, Zanthoxyli Radix, Aucklandiae Radix, Astragali Radix, Poria, Rehmanniae Radix, Paeoniae Radix Alba)	SJYY ^#^	+
JPACM-02-02	QZWTKL (Bupleuri Radix, Corydalis Rhizoma, Aurantii Fructus, Cyperi Rhizoma, Paeoniae Radix Alba, Glycyrrhizae Radix et Rhizoma Praeparata Cum Melle)	LNBXSY	+
JPACM-02-03	YWKL (Astragali Radix Praeparata Cum Melle, Codonopsis Radix, Citri Reticulatae Pericarpium, Cyperi Rhizoma, Paeoniae Radix Alba, Dioscoreae Rhizoma, Mume Fructus, Glycyrrhizae Radix et Rhizoma)	ZDQCBYY	+
JPACM-02-04	WKLJN (Paeoniae Radix Alba, Bletillae Rhizoma, Notoginseng Radix et Rhizoma, Glycyrrhizae Radix et Rhizoma, Poria, Corydalis Rhizoma, Sepiae Endoconcha, Belladonna Extract)	KHYY	+
JPACM-02-05	MRW (Cannabis Semen, Armeniacae Semen Amarum, Rhei Radix et Rhizoma, Aurantii Fructus Immaturus, Magnoliae Officinalis Cortex, Paeoniae Radix Alba)	NJTRT	+
JPACM-02-06	XYW (Bupleuri Radix, Angelicae Sinensis Radix, Paeoniae Radix Alba, Atractylodis Macrocephalae Rhizoma, Poria, Glycyrrhizae Radix et Rhizoma Praeparata Cum Melle, Menthae Haplocalycis Herba, Zingiberis Rhizoma Recens)	HNSWXZY	+
JPACM-02-07	XLJN (Scorpio, Bombyx Batryticatus, Sargassum, Scolopendr, Curcumae Radix, Prunellae Spica, Eupolyphaga Steleophaga, Laminariae Thallus Eckloniae Thallus, Agrimoniae Herba, Hirudo, Astragali Radix, Paeoniae Radix Alba, Pheretima, Hedyotidis Herba , Ostreae Concha)	JSSZXYJHYY	+

+: Detectable; −: Undetectable; ^*^: Abbreviated names of PAR-containing complex preparations; ^#^: Abbreviated names of drug companies

## 3. Experimental

### 3.1. Chemicals and Reagents

Methanol (HPLC grade) from Tedia Co., INC. (Fairfield, NJ, USA) and formic acid (analytical grade) from Nanjing Chemical Reagent Co. (Nanjing, China) were purchased. Ultrapure water was produced by a Milli-Q water purification system (Milford, MA, USA). The marker compound paeoniflorin was obtained from the National Institutes for Food and Drug Control (Beijing, China). Paeoniflorin sulfonate was isolated and identified from sulfur-fumigated PAR in our lab referring to the method of literature [6]. Their identities were confirmed by MS and NMR analysis, and the purity was determined to be higher than 95% by HPLC-UV analysis.

### 3.2. Plant Materials

Three batches of non-fumigated PAR were collected from Bozhou, Anhui province (JPACM-01-01), Shangqiu, Henan province (JPACM-01-03) and Jiang County, Shanxi province (JPACM-01-05), other commercial PAR samples were purchased from different pharmacy shops in China. All samples were authenticated by Prof. S.L. Li to be the root of *P. lactiflora* based on morphological and histological features according to the standards of Chinese Pharmacopoeia (2010 version) [1]. The PAR-containing complex preparations were purchased from different pharmacy shops in Nanjing, China (Table 1).

### 3.3. Sulfur-Fumigation of PAR

The sulfur-fumigated samples (JPACM-01-02, JPACM-01-04 and JPACM-01-06) were self-prepared in our lab from the non-fumigated samples (JPACM-01-01, JPACM-01-03 and JPACM-01-05) respectively following the modified procedures similar to that by herbal farmers or wholesalers: 100 g non-fumigated PAR samples were soaked with 10 mL water for 0.5 h, and 10 g sulfur powder was heated with an electric furnace until self-ignition, then the burning sulfur and wetted herbs were carefully put in the substratum and superstratum of a desiccator respectively. The desiccator was kept closed for 12 h. After that, the sulfur-fumigated PAR samples were taken out and dried at 40 °C for 12 h.

### 3.4. 50% Methanol Extracts of PAR and PAR-containing Complex Preparations

Pulverized non-fumigated, sulfur-fumigated PAR, commercial PAR and PAR-containing complex preparations were accurately weighed (approximately 0.2 g, or equivalent to 0.2 g PAR in the preparations) and ultrasonic-extracted with 10 mL 50% methanol for 25 min. The extracted solutions were filtered through a 0.22 μm PTFE syringe filter for HPLC-TQ-MS/MS analysis.

### 3.5. Water decoction of PAR

PAR powder (JPACM-01-02, about 1.0 g) was accurately weighed and refluxed with water (50 mL) for 30 min. The extract was then evaporated on a rotary evaporator until dry, the residue was dissolved with 50% methanol (50 mL), and the resultant solution was then filtered through a 0.22 μm PTFE syringe filter prior to injection into the HPLC-TQ-MS/MS system.

### 3.6. Liquid Chromatography

The HPLC analysis was performed on a Waters Alliance HPLC 2695 system (Waters Corp., Milford, MA, USA), equipped with a binary solvent delivery system, auto-sampler, and a photo-diode array (PDA) detector. The separation was achieved on a Waters Nova-Pak C18 analytical column (150 × 3.9 mm, 5 μm). The mobile phase consisted of (A) 0.1% formic acid in purified water and (B) methanol containing 0.1% formic acid. The gradient elution was optimized as follows: 10-30% B (0–20 min), 30–90% B (20–22 min), 90% B (22–30 min), 90–10% B (30–32 min), and 10% B (32–40 min). The flow rate was 1.0 mL/min and split to 0.2 mL/min for mass spectrometry analysis. The column and auto-sampler temperature were maintained at 35 °C and 10 °C, respectively. The injection volume was 20 μL. The monitoring UV wavelength was set at 270 nm, and the scan range for PDA was 190–400 nm.

### 3.7. Mass Spectrometry

Mass spectrometry was performed on a Micromass Quattro-Micro™ triple-quadrupole mass spectrometer (Waters Corp.) with electrospray ionization (ESI) interface in negative mode. For all the mass scan modes, the capillary voltage was 3,500 V, desolvation gas was set to 400 L/h at 400 °C, the cone gas was 50 L/h, and source temperature was set at 110 °C.

For full scan, the mass range was from m/z 200 to m/z 800. The cone voltages were set at 25 V and 40 V respectively. For daughter ion scans, the parent ions were m/z 449 and m/z 543. The mass range of daughter ion scan was from m/z 100 to m/z 600. The cone voltages were set at 25 V and 40 V for ions of m/z 449 and m/z 543, respectively, and the collision voltages were set at 15 V and 40 V, respectively. Argon was employed as the collision gas at a pressure of 4.0 × 10^−3^ mbar.

For MRM scans, the transitions m/z 449 → m/z 327, m/z 449 → m/z 165, m/z 543 → m/z 259 and m/z 543 → m/z 213 were simultaneously monitored. The cone voltages, collision voltages and argon pressure were set as the same as those in daughter ion scan mode.

The HPLC-TQ-MS/MS system was controlled, and the data were acquired and processed by Masslynx® version 4.1.

### 3.8. Sensitivity Test

For sensitivity test, 50% methanol stock solutions (1.0 mg/mL) of paeoniflorin and paeoniflorin sulfonate were diluted with 50% methanol to a series of concentrations, and analyzed with the MRM scan and UV/270 nm detection. The limit of detection (LOD) was defined as the lowest concentrations of paeoniflorin and paeoniflorin sulfonate which could be detected with a signal to noise ratio (S/N) of 3.

## 4. Conclusions

In this study, an HPLC-TQ-MS/MS with two mass transitions MRM scan method was developed for screening sulfur-fumigated PAR-containing complex preparations. Compared with previously reported LC-UV methods, this newly established method is more selective and sensitive for detecting the characteristic markers paeoniflorin and paeoniflorin sulfonate in sulfur-fumigated PAR-containing complex preparations, and should be a universal method for identifying sulfur-fumigated PAR in complex preparations with different constituent herbs.
